# Lipid traffic: an enigmatic alliance between ORPs and a TMEM16-like protein at membrane contact sites

**DOI:** 10.1042/BST20250365

**Published:** 2026-06-22

**Authors:** Alicia Fabbre, Guillaume Drin

**Affiliations:** Université Côte d'Azur, CNRS, INSERM, Institut de Pharmacologie Moléculaire et Cellulaire, 660 route des lucioles, 06560 Valbonne, France

**Keywords:** intrinsically disordered proteins, lipid scramblase, lipid transfer, membrane contact sites, phosphatidylserine, phosphoinositides

## Abstract

Lipid transfer proteins (LTPs) play a critical role in distributing lipids within eukaryotic cells. In yeast, Osh6 and Osh7, which belong to the oxysterol-binding protein-related protein family, transfer phosphatidylserine (PS) from the endoplasmic reticulum (ER) to the plasma membrane (PM) in exchange for phosphatidylinositol 4-phosphate (PI(4)P). These proteins localize at ER–PM contact sites by associating with Ist2, an ER-resident TMEM16-like protein that bridges the ER and PM via a long intrinsically disordered region (IDR). Recent studies have shown that this association ensures accurate PS transfer by concentrating Osh6 and Osh7 at the ER–PM interface while preserving their ability to access both membranes. However, it remains unclear how these LTPs function when bound to the Ist2 IDR, whose length far exceeds the ER–PM distance at contact sites, and why they do not integrate both the tethering and the PS/PI(4)P exchange functions, like their human homologs. Additionally, it has been revealed that Ist2 can transfer lipids across the ER membrane via a scramblase activity. Yet, whether and why this activity is coupled to the PS/PI(4)P exchange activity of Osh6 and Osh7 remains unknown. The Ist2–Osh6/7 system emerges as a fascinating model that integrates tethering, scramblase, and lipid exchange functions. Future studies of this system are likely to provide important insights into how lipid transfer processes are coordinated at membrane contact sites.

## Introduction

Eukaryotic cells have a compartmentalized architecture based on membranes with defined features, composed of a high diversity of lipids and proteins [1]. Lipids self-organize into bilayers to form these membranes and exert signaling functions through interactions with specialized proteins [2,3]. To fulfill these roles, lipids are precisely distributed within the cell [4–6]. A remarkable example is phosphatidylserine (PS), an anionic phospholipid. In yeast and human cells, PS is scarce in the endoplasmic reticulum (ER) and most organelles, yet it is highly abundant—accounting for up to 30% of lipids—in the inner leaflet of the plasma membrane (PM), where it contributes negative charge [7]; this is critical for recruiting signaling proteins (e.g., K-Ras and MARCKS) via electrostatic interactions [3,8].

**Figure 1:**
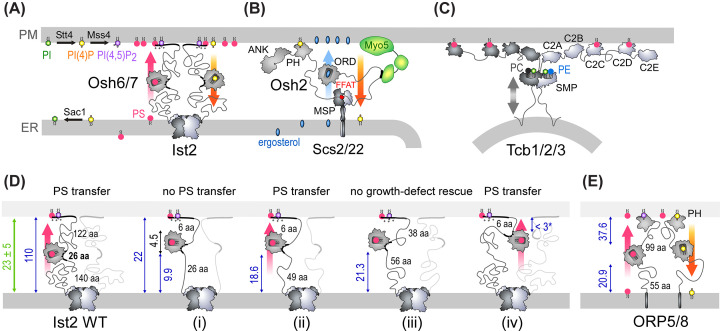
Ist2–Osh6/7 system and other key tethering factors involved in lipid transfer processes at ER–PM contact sites (**A**) PS, synthesized in the ER, is transferred by Osh6/7 to the PM by PS/PI(4)P exchange. The phosphorylation of PI into PI(4)P by Stt4 at the PM, and PI(4)P hydrolysis by Sac1 at the ER, maintain a PI(4)P concentration gradient that drives exchange cycles and the accumulation of PS in the PM. Ist2 has a transmembrane domain (TMD) embedded in the ER membrane and a long IDR whose C-terminal polybasic motif can interact with PS and PI(4,5)P_2_ (phosphatidylinositol 4,5-bisphosphate) in the PM, thereby allowing Ist2 to form ER–PM contact sites. PI(4,5)P_2_ is generated by the phosphorylation of PI(4)P by Mss4. Osh6/7 interact with a central motif of the Ist2 IDR to concentrate in the ER–PM contact sites and transfer PS and likely PI(4)P between the membranes accurately. (**B**) Osh2 and Osh3 (omitted for clarity) localize to ER–PM contact sites by associating via their PH domain to PI(4)P in the PM and via their FFAT motif to the MSP (Major Sperm Protein) domain of ER-resident Scs2/22 proteins. Osh2 additionally binds to Myo5 to localize to endocytic sites and deliver there ergosterol, synthesized in the ER, by sterol/PI(4)P exchange mediated by its ORD. The function of its ankyrin repeat domain (ARD) is unknown. Note that Osh2 is localized at the rims of ER–PM contact sites [22]. (**C**) Tricalbins Tcb1/2/3, anchored to the tubular ER via hydrophobic hairpin motifs, form heterodimers via their SMP domain and associate with the PM via C2 domains that bind to PS (it is unclear which C2 domains bind to the PM, except for Tcb3 [24]). Like other SMP domain-containing LTPs [13], tricalbins should transfer phospholipids (PC, PE, PI, PS) with weak selectivity. (**D**) The Osh6/7 transfer activity depends on the Ist2 IDR length and the position of their binding motif within the IDR. The segment of the Ist2 IDR crossing the ER–PM gap consists of the Osh6/7-binding site (26 aa) flanked by two regions of 122 and 140 aa [30], considering that the [878–946] region of the Ist2 IDR, encompassing the polybasic motif [34,74], binds to the PM. This segment is considered to have a maximal end-to-end length *L =* 110 nm when it adopts a fully extended conformation (considering 0.38 nm *per* residue [75], *L* = 0.38 × *n* and *n* = 288): it exceeds the average ER–PM distance at contact sites. PS transfer assays show that Osh6 is inefficient in yeast expressing a ‘short’ Ist2 construct (i) whose IDR segment between its TMD and its PM-binding segment comprises 58 aa, because the Osh6/7-binding site cannot be more than 10 nm away from the ER; Osh6/7 (4.5 nm diameter) cannot reach the PM. PS transfer is restored with a slightly longer construct (ii), as the Osh6/7-binding site can be 18 nm above the ER. However, growth-rescue assays suggest that Osh6/7 are not functional if their binding site in the Ist2 IDR is 21 nm above the ER (iii). PS transfer assays indicate that Osh6/7 remain functional even if their binding site is close to the PM, likely because the association of the IDR with the PM is dynamic and does not impose constraint (iv). The lengths of Ist2 IDRs, considered in a fully extended conformation and expressed in nanometers, are indicated in blue. The ER–PM distance at contact sites in yeast is indicated in green. For construct (iv), note that the length marked with an asterisk between the Osh6/7-binding site and the PM is likely underestimated because it is unclear if the [878–946] segment is entirely bound to the PM. (**E**) ORP5/8 integrate a membrane tethering function with a PS/PI(4)P exchange activity. Structurally, they resemble the Ist2–Osh6/7 system with two IDRs connecting the ORD to the ER and PM. The total length of the intrinsically disordered part of ORP5/8 (indicated in blue only for ORP8) largely exceeds the intermembrane gap of the ER–PM contact site.

Like most lipids and lipid precursors in eukaryotic cells, PS is synthesized in the ER. The mechanism by which it is exported and enriched in the PM remained unknown until the discovery that, in *Saccharomyces cerevisiae*, Osh6 and Osh7—two members of the oxysterol-binding protein (OSBP)-related protein (ORP) family—mediate this process [9]. Osh6/7 consist of an OSBP-related domain (ORD) with a pocket to specifically host a molecule of either PS or phosphatidylinositol 4-phosphate (PI(4)P) [9,10]. PI(4)P is a lipid belonging to the phosphoinositide class that is mainly synthesized in the Golgi membrane and the PM through the phosphorylation of phosphatidylinositol (PI) [6]. It was therefore proposed that Osh6/7 act as PS/PI(4)P exchangers to accumulate PS in the PM: they extract PS from the ER, exchange PS for PI(4)P at the PM, and transfer PI(4)P to the ER. PI(4)P is then hydrolyzed into PI by the ER-resident enzyme Sac1, enabling Osh6/7 to extract a new PS molecule (Figure 1A). This model was validated by experiments showing that PI(4)P synthesized at the PM by Stt4 must be ‘consumed’ by Sac1 to maintain a PI(4)P concentration gradient at the ER–PM interface, driving continuous Osh6/7-mediated exchange cycles and the build-up of PS in the PM [10].

Other lipids are transported intracellularly by ORP/Osh proteins or by structurally unrelated proteins, collectively known as lipid transfer proteins (LTPs) [11–14]. Most LTPs localize to membrane contact sites, where two organelle membranes are separated by 5–50 nm—a very short distance compared with the whole-cell dimensions [14,15]. The PM is also engaged in contact sites, particularly with the ER. In yeast, ER–PM contact sites are mainly stabilized by six tethering factors that are also involved in lipid transfer [16–18]. Scs2 and Scs22, anchored to the ER via a transmembrane segment, associate with Osh2 and Osh3 to bridge the ER and PM [19–22]. These Osh proteins are more sophisticated than Osh6/7, as they comprise, in addition to an ORD with an ergosterol/PI(4)P exchange or PI(4)P transfer capacity, a PH (Pleckstrin Homology) domain for PM binding and a short FFAT (two phenylalanines (FF) in an acidic tract) motif recognized by Scs2/22 [23] (Figure 1B). Tricalbins (Tcb1/2/3), anchored to the ER via an N-terminal hydrophobic hairpin, bind to the PM through C2 domains and transfer lipids via an SMP (synaptotagmin-like mitochondrial lipid-binding) domain [16–18,24] (Figure 1C). Together, these proteins are essential for maintaining the complex lipid composition and organization of the PM, thereby ensuring both its integrity and functional features [17,22,25].

Ist2, the sixth key tethering factor involved in establishing ER–PM contact sites, was found to recruit Osh6/7 (Figure 1A) [26,27]. This explained why these LTPs localize to these contact sites, despite lacking known membrane-targeting domains [28]. This association was next found to be essential for PS delivery to the PM and for amino phospholipid metabolism, as PS undergoes endocytosis and is converted into phosphatidylethanolamine (PE) in the endosomal compartment [26,27]. Recent findings clarify why Osh6/7 must bind Ist2 to be active and show that Ist2 is not only a tether but also a phospholipid scramblase [29–31]. These discoveries raise new questions about how the Ist2–Osh6/7 complexes function at ER–PM contact sites and regulate lipid fluxes across and between cell membranes.

## Ist2 concentrates Osh6 at contact sites for accurate PS transfer

Ist2 is an ER-resident transmembrane protein with an intrinsically disordered region (IDR) of ∼360 amino acids (aa) exposed to the cytosol [32]. This long and flexible region associates with the PM via a C-terminal polybasic motif that binds PS and the phosphoinositide PI(4,5)P_2_ via electrostatic interactions, enabling Ist2 to form ER–PM contact sites (Figure 1A) [33,34]. The Čopič team and later the Loewen team reported that Osh6/7 localize to these contact sites as they bind to a central motif within the Ist2 IDR [26,27]. This interaction is critical: mutations that disrupt the Osh6:Ist2 interaction severely impair Osh6 transfer activity [26,27]. Structural investigations and AI-guided molecular modeling show that Osh6 binds the Ist2 IDR via a surface region distal to the entrance of its lipid-binding pocket [29,30]. This indicates that Osh6/7 could transfer lipids while associated with Ist2; however, why must they be associated with Ist2 to be efficient?

Several yet non-exclusive assumptions are commonly made about why LTPs are at contact sites. One hypothesis, rather disputed, is that this enables ultrafast lipid transfer, as the short intermembrane distance at contact sites would substantially reduce the time LTPs must shuttle between membranes [35,36]. A second hypothesis is that restricting LTPs to contact sites prevents misdelivery of lipids to other (and incorrect) organelles, enhancing transport accuracy [35,36]. A third idea, experimentally more supported, is that colocalization of LTPs at contact sites enables coordination of lipid fluxes between membranes [37–39]. With these notions in mind, we examined why Osh6 becomes efficient upon binding to the Ist2 IDR at artificial ER–PM contacts in a setup that mimics intracellular compartmentalization.

To reconstitute contact sites, we attached the N-terminal end of a purified form of Ist2 IDR to liposomes (ER-mimetic), which were next connected to PM-mimetic liposomes rich in PS and PI(4,5)P_2_. We then established that Osh6 specifically and quickly transferred PS between these connected liposomes in the presence of a third, free population of liposomes. In the absence of Ist2 IDR, Osh6 slowly and evenly distributed PS from the ER-mimetic liposomes to all the others [30]. These data suggest that the poor PS enrichment in the yeast PM, resulting from the absence of Ist2 or its inability to recruit Osh6/7 [26], correlates with a slow transfer of PS to the PM and its potential redistribution to other intracellular sites, as Osh6/7 are fully cytosolic. It is unclear whether these LTPs also associate with Ist2 to transfer PI(4)P accurately, but this is likely, given data suggesting that they supply the ER with PI(4)P specifically originating from the PM [16,40–42]. Collectively, these findings suggest that Ist2 ensures focalized, fast PS/PI(4)P exchange within the cell's complex interior by spatially restricting Osh6/7 at ER–PM contact sites.

Importantly, unlike LTPs that both connect membranes and transfer lipids, the Ist2–Osh6/7 system is unique in that these two functions can be separated. This allowed us to show that an Osh6 mutant, unable to associate with the Ist2 IDR, weakly transferred PS between membranes connected by this segment [30]. This is consistent with the observation that PS transfer is impaired when Osh6/7 cannot be recruited by Ist2, even though the ER–PM contact sites are intact [26]. These results suggest that LTPs function optimally at contact sites because they are concentrated between membranes, not because membranes are close.

## The length of the Ist2 IDR impacts Osh6/7 transfer activity

Considering that the segment of the Ist2 IDR crossing the ER–PM gap consists of the Osh6/7-binding site (26 aa) flanked by two regions of 122 and 140 aa [30], we can estimate that its length could reach up to 110 nm if it adopts a fully extended conformation (Ist2 WT, Figure 1D). This greatly exceeds the typical ER–PM distance observed at contact sites (16–34 nm, with an average of 23 ± 5 nm [17]). The Čopič team analyzed how shortening this segment affects Osh6 activity using *in cellulo* PS transfer assays under conditions in which Ist2 still forms contact sites and recruits Osh6 [30]. Reducing this segment to 58 aa abolishes Osh6 activity (construct (i), Figure 1D), even though its length (estimated at ~22 nm if fully extended) is sufficient to span the ER–PM gap. However, one can estimate that Osh6 is confined to an action range of ∼10 nm above the ER. Even accounting for its diameter (4.5 nm), it cannot reach the PM, which explains its loss of activity. PS transfer is restored when the Osh6/7-binding site is positioned ∼19 nm away from the ER (construct (ii), Figure 1D). This parallel analyses demonstrating that Ysp2, which belongs to the Lam (LTP anchored at membrane contact sites) family in yeast [43], cannot move sterols at ER–PM contact sites if its START-like transfer module is tethered to the ER by an IDR shorter than 40 aa (∼15 nm) [44]. These findings are also consistent with *in vitro* analyses showing that E-Syt1 (mammal tricalbin orthologs) cannot transfer lipids between membranes separated by more than the length of the linker anchoring its SMP domain to one membrane [45]. However, the Dutzler team obtained contrasting results using growth-rescue assays with *ist2;psd1* knockout strains and different Ist2 constructs, suggesting that Osh6/7 no longer transfer PS, although they can be localized ∼21 nm from the ER surface, according to our estimations ( [29]construct (iii), Figure 1D). All these results, albeit quantitatively different, support the idea that Osh6/7, when bound to Ist2, do not properly function if they have a lower capacity to reach both the ER and the PM.

Additionally, the Dutzler and Čopič teams investigated the effects of preserving the native length of the Ist2 IDR while repositioning the Osh6/7-binding site—either near its N-terminal end or near its PM-binding segment [29,30]. The first team observed that this severely affects Osh6 activity in both cases. In contrast, the second team found that Osh6 still functions normally when its binding site is close to the PM (<3 nm, construct (iv), Figure 1D). One can propose that the C-terminal segment of the IDR imposes fewer constraints on Osh6/7 movement than the N-terminal segment, possibly because the former binds dynamically to the PM (in contrast, the latter is stably anchored to the ER via the Ist2 TMD). Further analyses are needed to reach a consensus on how the position of the Osh6/7-binding site along the Ist2 IDR affects lipid transfer.

Finding that Osh6/7 activity strongly depends on the location of their binding site along the Ist2 IDR is puzzling, given the reversible nature of the Ist2:Osh6/7 association. This suggests that Osh6/7 perform multiple lipid-transfer cycles while remaining associated with Ist2. It also underscores that while the Ist2 IDR concentrates Osh6/7 at the ER–PM contact site, it must preserve their ability to access membranes to extract and deliver lipids, thereby ensuring efficient lipid transfer.

## Questions on the recruitment of Osh6/7 by the Ist2 IDR

Because the length of the Ist2 IDR, when considered in an extended conformation, largely exceeds the average ER–PM gap at contact sites, one can wonder whether the Ist2 IDR dynamically adopts different compact conformations to fit within this narrow intermembrane space and how Osh6/7 transfers lipids through this crowded environment. One corollary question is why the Ist2 IDR is so long. Mathematical modeling suggests that Osh6/7 should encounter significant mechanical resistance once the Ist2 IDR is slightly shortened, preventing their access to the ER and the PM [29]. However, this model is challenged by the data showing that Osh6 efficiently transfers PS when connected to a very shortened IDR [30]. We can therefore assume that the length of the Ist2 IDR confers other functional and potentially regulatory advantages. As observed for OSBP [46], it might act, by dynamically occupying a large volume, as an entropic shield, limiting the local density of Osh6/7 or regulating their capacity to move in and out of contact sites. Another possibility is that it helps Ist2, and thus Osh6/7, to segregate from other tethering factors and LTPs at contact sites, as observed in yeast [18], maybe to both control ER architecture and locally fine-tune the lipid composition of the PM. It is also possible that the Ist2 IDRs form condensate via liquid-liquid phase separation in the 2D-like environment of contact sites via multiple weak intermolecular interactions, as found for the LTP PDZD8 [47]; this might allow Ist2 to act as a ‘glue’ able to efficiently bridge the ER and the PM via interfacial tension. One can also envisage that, due to its length, the Ist2 IDR adopts many conformations, whose occurrence may be regulated by post-translational modifications (e.g., phosphorylation [48]) to regulate lipid transfer. Finally, the length of the Ist2 IDR may indicate the presence of binding sites for additional protein partners. Further work is necessary to examine whether the Ist2 IDR performs multiple, non-exclusive, and regulatable functions, ranging from the control of Osh6/7 mobility to the establishment of distinct areas at the ER–PM contact sites and possibly the control of how PS and PI(4)P are distributed at the nanoscale level in the PM [49].

Interestingly, Osh6/7, when associated with the Ist2 IDR, resemble their closest homologs, ORP5/8, which function as PS/PI(4)P exchangers at ER–PM contact sites in human cells [50]. These comprise a central ORD connected via two long IDRs to an N-terminal PH domain and a C-terminal transmembrane segment, which are respectively anchored to the PM and the ER (Figure 1E). Thus, the data obtained from the Ist2–Osh6/7 system may offer valid conclusions on the functional roles of IDRs in ORP5/8 and other complex LTPs [51]. Intriguingly, growth-rescue assays suggested that Osh6—when directly inserted into the middle of the Ist2 IDR—properly transfers PS to the PM [29]. This raises the question of why yeast cells do not possess a direct equivalent of ORP5/8, which combines both lipid-exchange and membrane-tethering activities [50]. Maybe the capacity of Osh6/7 to be partially cytosolic, due to their relatively low affinity (K_D_ ∼ 1–10 μM) for Ist2 [29,30], offers functional advantages. There is no evidence that Osh6/7 exert a PS transfer activity outside ER–PM contact sites to, for instance, support PS decarboxylation in mitochondria or provide PS to secretory vesicles involved in the polarized accumulation of PS in the PM during yeast budding and division [9,52]. However, Osh6 can substitute for Osh4, a sterol/PI(4)P exchanger that removes PI(4)P from secretory vesicles to promote their fusion with the PM [53]. Osh6 also restores growth of yeast lacking Osh4 and all the proteins involved in scaffolding the ER–PM contact sites, i.e., Tcb1/2/3, Scs2/22, and Ist2 [25]. We can thus presume that some Osh6/7 functions outside contact sites, in an Ist2-independent manner, perhaps to down-regulate PI(4)P levels on vesicles circulating in the cytosol. If true, they could therefore function differently from ORP5/8, which transfer lipids to non-PM compartments (mitochondria and lipid droplets) but remain at contact sites to exert this alternative activity due to their permanent attachment to the ER [54,55]. Finally, in-cell, biochemical, and structural investigations suggest that Osh6/7 constitutively associate with Ist2 to localize at contact sites [26,27,29,30]. Nevertheless, the Osh6/7-binding motif of the Ist2 IDR contains two key threonine residues [26,29,30] whose substitution with phosphomimetic ones was found to reduce the association of Ist2 with Osh6 [26]. Thus, the separation of lipid-exchange and membrane-tethering activities into distinct proteins may also provide a mechanism to regulate PS transfer to the yeast PM via a phosphorylation-dependent process.

## Ist2 is an ER phospholipid scramblase

Ist2 TMD was known to share similarities with certain TMEM16 proteins that can powerfully accelerate the passage of phospholipids between the two leaflets of cell membranes—a process called lipid scrambling [56–58]. This suggested that Ist2 might also have this function, though an initial study in which it was reconstituted into proteoliposomes challenged this possibility [59]. Recently, the Lenoir and Čopič teams, in collaboration, along with the Dutzler team, possibly as they used other reconstitution protocols, have found that Ist2 does indeed act as a lipid scramblase [29,31]. Furthermore, cryo-EM studies and molecular dynamics simulations revealed that the TMD of Ist2 dimerizes and resembles TMEM16 scramblases [29,31], with a continuous polar groove between α-helices 4 and 6 that allows passage of the lipid headgroup between leaflets, following the ‘credit card’ model [60]. Most known TMEM16 require calcium (Ca^2+^) to function, and this is structurally related to the coordination of Ca^2+^ by five conserved residues that induce the rigidification of α-helix 6 and the opening of the groove [60]. In contrast, Ist2 has a Ca^2+^-independent, constitutive scrambling activity due to the substitution of three of these residues, which introduces intrachain salt bridges that maintain the groove permanently open [29]. Molecular dynamic simulations predicted that Ist2 scrambles phospholipid species with some selectivity (phosphatidylcholine (PC) > PS > PE > PI > phosphatidic acid (PA)) [31]. Nevertheless, *in vitro* assays showed that Ist2 can scramble various phospholipids, notably PS, without preference [31].

The ER is a biogenic membrane that must expand rapidly. However, phospholipids are synthesized in only one leaflet of the ER membrane. To ensure symmetrical phospholipid distribution and support membrane expansion, these lipids must be rapidly translocated to the other leaflet. In this context, identifying Ist2 as an ER-resident scramblase is of first importance. Ist2 has been further shown to influence membrane-remodeling processes—such as COPII vesicle formation and lipid droplet biogenesis—by regulating lipid balance between the ER leaflets [31].

## Questions on the coupling between the lipid transport activities of Ist2 and Osh6/7

**Figure 2:**
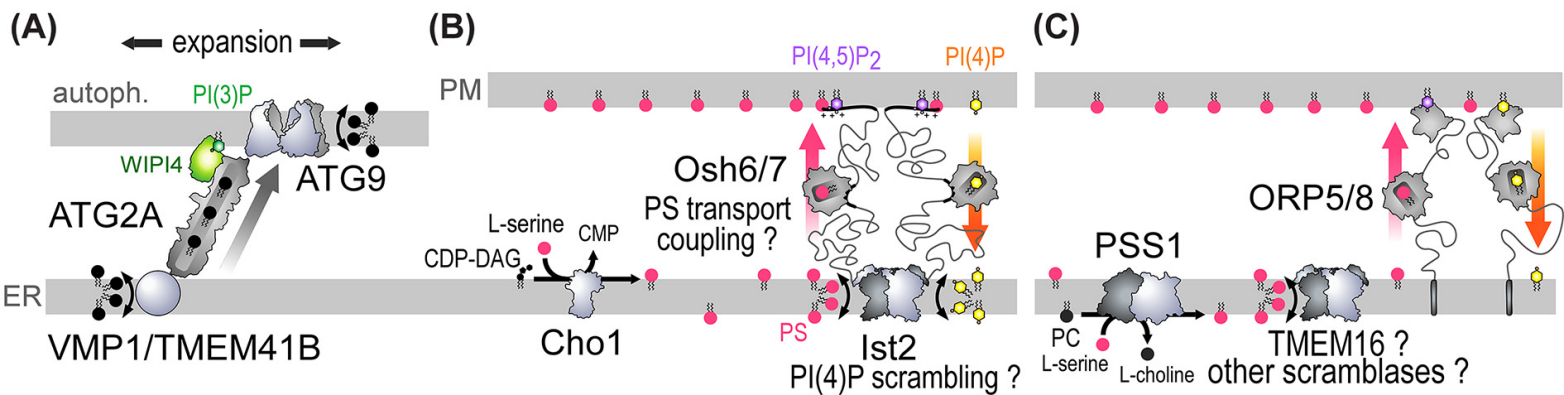
Questions on the functional interplay between scramblases and ORP/Osh proteins (**A**) The ATG9 scramblase, in a trimeric form, and scramblases of the DedA family (VMP1 and TMEM41B, whose structure is unknown), cooperate with the BLTP ATG2A to ensure the expansion of the autophagosome membrane via a massive delivery of phospholipids produced in the ER. The role of the scramblases would be to re-equilibrate lipids between the two leaflets of the ER and autophagosomal membranes to maintain their lamellar structure. Note that this system is structurally very different from the Ist2–Osh6/7 system and serves a different function (building a membrane, rather than fine-tuning its lipid composition). (**B**) The Ist2 transmembrane domain closely resembles TMEM16 proteins and scrambles phospholipids, including PS. It is not known whether the PS transport activities of Osh6/7 and Ist2 are coupled in yeast cells. Because Osh6/7 substitute PS with PI(4)P molecules in the ER during PS/PI(4)P exchange cycles, the Ist2’s scramblase activity is likely unnecessary to correct a lipid number imbalance between the ER leaflets. As Cho1 presumably produces PS from cytidine diphosphate (CDP)-DAG in the cytosolic leaflet of the ER, it is also unlikely that its activity would support Osh6/7 access to PS. Another question is whether Ist2 can scramble PI(4)P. (**C**) In human cells, PS is synthesized in the lumenal leaflet of the ER; whether ER-resident scramblase(s) move PS between the ER leaflets to regulate ORP5/8 is unknown.

That Ist2 can move PS across membranes *in vitro* suggests that it may also specifically regulate Osh6/7's capacity to transfer PS between membranes. Consistent with this, evidence suggests that other scramblases associate with LTPs termed bridge-like LTPs (BLTPs) to facilitate lipid transfer [14]. BLTPs are giant, rod-shaped proteins traversed by a long hydrophobic channel that is assumed to transfer phospholipids in a massive, non-selective manner at contact sites [14]. A well-studied example is ATG2, which transports phospholipids synthesized in the ER to a structure called the autophagosome to promote its expansion [61] (Figure 2A). The autophagosome eventually seals itself, engulfing cytosolic material before delivering it to the lysosomal compartment for degradation. ATG2 interacts with two ER scramblases, TMEM41B and VMP1, as well as a third, ATG9, localized in the autophagosomal membrane [62–66]. A popular hypothesis among cell biologists suggests that, as BLTPs extensively extract lipids from the cytosolic leaflet of the ER to deliver them to the cytosolic leaflet of an acceptor membrane, scramblase activity is required to keep a similar number of lipids between the leaflets of each membrane. This process would alleviate tensions caused by lipid flux and maintain membrane integrity. However, this hypothesis is unlikely to hold for the Ist2–Osh6/7 system.

Indeed, Osh6 and Osh7 are lipid exchangers: for each PS molecule they extract from the ER cytosolic leaflet, they supply one PI(4)P molecule in return. This cannot create an imbalance between the ER leaflets—at least in terms of lipid number (Figure 2B). Therefore, an alternative hypothesis, given that PS is synthesized in a single ER leaflet, is that Ist2 regulates the pool of PS available for Osh6/7 through its scrambling activity. Supporting this idea, we found that Ist2 reconstituted into proteoliposomes could, via its scramblase activity, sustain PS transfer by Osh6 to PM-mimetic liposomes by continuously replenishing the outer leaflet of the proteoliposomes with PS from the inner leaflet [30]. This revealed that a scramblase and an LTP can cooperate to transfer a specific lipid species across and between membranes. However, there is no evidence yet that such coupling occurs in cells.

In yeast, PS is symmetrically distributed between the ER leaflets [67]. It is produced by Cho1, which transfers L-serine to the phosphatidyl group of CDP-diacylglycerol. Considering its close resemblance to another CDP-alcohol phosphatidyltransferase, PI synthase, whose catalytic site faces the cytosol [68], Cho1 likely produces PS in the ER cytosolic leaflet. Thus, Ist2 appears dispensable for generating a PS pool in this ER leaflet to support Osh6/7 function. Consistent with this, Osh6/7-mediated PS transfer and PS homeostasis are barely affected in yeast cells expressing an Ist2 variant that only comprises the IDR and the last two helices of the scramblase domain [31].

In mammalian cells, PS is likely generated in the ER lumenal leaflet, as structural analyses indicate that the catalytic sites of human PSS1 and PSS2, which synthesize PS by respectively exchanging PC or PE headgroups with L-serine, face the ER interior [69,70]. How newly made PS is subsequently distributed within the ER remains enigmatic. Contrasting reports indicate that PS is mainly localized to the luminal or cytosolic leaflet of the ER [67,71]. Some evidence suggests that the scramblases TMEM16K, but also TMEM41B, and VMP1 impact the transbilayer PS distribution in the ER [67,72]. Whether these scramblases modulate the activity of ORP5/8 and ORP10, a PS/PI(4)P exchanger at ER-endosome contact sites [73], is unknown (Figure 2C). Overall, whether Ist2 and other ER-resident scramblases regulate ORP/Osh-mediated PS transfer warrants further investigations.

Our picture of the Ist2–Osh6/7 partnership is further complicated by recent data showing that a pool of PI(4)P synthesized by Stt4 in the cytosolic leaflet of the PM is exposed to the other side of this membrane in yeast cells that do not express the Golgi-resident lipid flippase Neo1 [42]. Additional evidence suggests that PI(4)P is transferred by Osh6 (and likely Osh7) from the PM to the ER cytosolic leaflet, escapes degradation by Sac1, reaches the ER and Golgi lumenal leaflets, and is then redistributed to the cytosolic leaflet of the Golgi via the Neo1 flippase activity. This suggests that Ist2 transfers PI(4)P via its scrambling activity to the lumenal ER leaflet after Osh6 delivers it. So far, there is only a few data suggesting that Ist2 affects cellular PI(4)P levels [16]. Thus, further investigations are needed to elucidate why PI(4)P follows this particular lumenal ER-to-Golgi route and whether this is linked to the combined action of Ist2 and Osh6/7 transport activities.

Interestingly, tricalbins Tcb1/2/3, upon forming ER–PM contact sites, locally induce high positive curvature in the ER membrane [17,18]. This creates lipid packing defects in the ER membrane, thereby facilitating lipid extraction and their transfer to the PM. In contrast, Ist2 is largely localized in ER sheets, whose membranes are flat [17,18]. It can thus be interesting to examine whether Ist2 maintains the geometry of these ER regions by equilibrating lipids between ER leaflets, thereby preventing curvature generation. It is also interesting to examine whether the primary role of Ist2 scrambling activity may be to locally disorganize lipids, thereby generating lipid packing defects that facilitate Osh6/7-mediated PS extraction [29].

## Concluding remarks

The Ist2–Osh6/7 system is remarkable, as it combines membrane tethering, lipid scramblase, and lipid exchange functions. Further investigations are needed to elucidate how Osh6/7 function in the crowded environment of the contact site when associated with Ist2 and to determine whether the reversibility of the Ist2:Osh6/7 interaction confers functional advantages. Furthermore, it is essential to fully understand whether Ist2’s scramblase activity is coupled to Osh6/7-mediated transfer in cells, notably given recent observations that Osh6 and Neo1 are functionally linked in regulating PI(4)P topology. Despite its singularity, the Ist2–Osh6/7 system shares features with sophisticated LTPs that exhibit dual tethering/transferring activities, as well as with molecular machineries in which lipid scrambling supports massive lipid transfer between membranes. Therefore, studying the Ist2–Osh6/7 system should provide general insights into how LTPs and scramblase-LTP systems function at membrane contact sites.

## Perspectives

- Understanding how membrane tethering, lipid transfer, and scrambling are coordinated at membrane contact sites is essential to explain how lipids are distributed within the cell.
- Recent studies explain why in yeast the tethering factor Ist2 supports PS transfer at ER–PM contact sites by recruiting Osh6/7 via its long, cytosolic IDR and unveil that Ist2 has a lipid scramblase activity that impacts ER functions.
- Key challenges include determining how the Osh6/7, bound to the Ist2 IDR, functions in the crowded environment of contact sites and whether Ist2 scramblase activity is coupled to Osh6/7-mediated lipid exchange in cells.

## Open Access

Open access for this article was enabled by the participation of INSERM in an all-inclusive Read & Publish agreement with Portland Press and the Biochemical Society.

## Abbreviations

aa: amino acid
AI: artificial intelligence
BLTP: bridge-like LTP
CDP: cytidine diphosphate
ER: endoplasmic reticulum
IDR: intrinsically disordered region
Lam: LTP anchored at membrane contact sites
LTP: lipid transfer protein
ORD: OSBP-related domain
ORP: oxysterol-binding protein-related-proteins
Osh: oxysterol-binding homology
PC: phosphatidylcholine
PE: phosphatidylethanolamine
PH: Pleckstrin Homology
PI: phosphatidylinositol
PI(4)P: phosphatidylinositol 4-phosphate
PI(4,5)P_2_: phosphatidylinositol 4,5-bisphosphate
PM: plasma membrane
PS: phosphatidylserine
SMP: synaptotagmin-like mitochondrial lipid-binding
TMD: transmembrane domain
TMEM: transmembrane protein
